# Direct Oral Anticoagulants versus Vitamin K Antagonists for the Treatment of Left Ventricular Thrombosis: A Meta-Analysis

**DOI:** 10.31083/j.rcm2309312

**Published:** 2022-09-13

**Authors:** Jian Li, Yuecheng Hu, Zhenhua Wu

**Affiliations:** ^1^Intensive Care Unit, Tianjin Chest Hospital, 300222 Tianjin, China; ^2^Department of Cardiology, Tianjin Chest Hospital, 300222 Tianjin, China

**Keywords:** vitamin K antagonists, left ventricular thrombosis, direct oral anticoagulants, meta-analysis

## Abstract

**Background:**

Vitamin K antagonists (VKAs) have been recommended as first-line anticoagulants for patients with left ventricular thrombosis (LVT). Direct oral anticoagulants (DOACs) are used as an alternative to the standard of care in anticoagulation. The aim of this meta-analysis was to compare the efficacy and safety of VKAs and DOACs in the treatment of patients with LVT.

**Materials and Methods:**

Studies were identified by searching the PubMed, Web of Science, and Embase. The main outcomes included stroke or systemic embolism (SSE), thrombus resolution, and bleeding events. The pooled risk ratio (RR) with 95% confidence intervals (CIs) was estimated with fixed effect or random effect models.

**Results:**

Seventeen studies were included. Pooled estimate showed that DOACs had comparable efficacy in prevention of SSE (RR = 0.96, 95% CI: 0.80, 1.16; *p* = 0.677) and thrombus resolution as compared with VKAs (RR = 1.07, 95% CI: 0.97, 1.18; *p* = 0.193). DOACs significantly decreased the risk of stroke in patients with LVT (RR = 0.68, 95% CI: 0.47, 1.00; *p* = 0.048). However, this effect was not observed in the sensitive analysis by high-quality studies (RR = 0.69, 95% CI: 0.47, 1.02; *p* = 0.06). In terms of safety outcomes, DOACs had similar risk of bleeding events (RR = 1.12, 95% CI: 0.80, 1.57; *p* = 0.386) and clinically relevant bleeding events (RR = 0.49, 95% CI: 0.23, 1.03; *p* = 0.060). Meta-regression analysis demonstrated that none of the variables (study design, concomitant antiplatelet medication, duration of follow-up, primary cause of LVT, sample size, types of DOACs) had an impact on the risk of SSE, thrombus resolution and bleeding events. Subgroup analysis based on the use of antiplatelet and treatment switching revealed that there were no significant differences among patients with different treatment regimens.

**Conclusions:**

Based on the present evidence, both DOACs and VKA offered similar effective and safe outcomes in patients with LVT.

## 1. Introduction

Left ventricular thrombus (LVT) is a frightening complication occurring in patients with acute myocardial infarction (MI), heart failure, and various cardiomyopathies [1, 2]. The estimated incidence of LVT ranges from 15% to 25% in patients with anterior [1] and 36% in patients with dilated cardiomyopathy when optimal imaging modalities are used [3]. LVT has been found to increase risk of stroke, systemic embolism, and subsequent morbidity and mortality [4]. In patients with LVT after acute MI, most thromboembolic events occur within the first 4 months [5], whilst in most cases, thrombus is no longer visible within 3–6 months [6].

Vitamin K antagonist (VKA) is recommended as first-line therapy for at least 3 months in patients with LVT, on the basis of the risks of thrombus resolution and individual bleeding [7]. However, the direct oral anticoagulants (DOACs) have attracted great attention for the treatment of LVT since they have consistent anticoagulant effect and have no dietary restrictions or monitoring for international normalized ratio (INR) [8, 9]. Moreover, they also have decreased the incidence of intracranial bleeding as compared to VKAs [10, 11, 12, 13].

Several studies have compared the effect and safety profiles of DOACs with VKAs in patients with LVT. However, their results remain controversial because of the scarce data [8, 9]. Therefore, the aim of this study was to provide reliable evidence for the efficacy and safety comparison between DOACs and VKAs in patients with LVT.

## 2. Methods

### 2.1 Data Sources and Search Strategy

This study was conducted following the Preferred Reporting Items for Systematic Reviews and Meta-analysis (PRISMA) statement guidelines [14]. On August 2, 2021, we searched for relevant articles in Embase, PubMed and Web of Science. The literature search was last updated on April 10, 2022. The search utilized the following terms: (left ventricular thrombus OR left ventricular thrombi, OR intracardiac thrombus) AND (anticoagulation OR anticoagulants OR direct oral anticoagulants OR DOAC OR NOAC) AND (vitamin K antagonists). The search involved human subjects, and had no imposes on language. The reference citations of included articles were also searched to include more relevant studies.

### 2.2 Study Selection

Inclusion criteria were as followings: (1) study design: case-control study, randomized control trial (RCT), cohort, or comparative study; (2) study objects: patients diagnosed with LVT based on appropriate cardiac imaging techniques; (3) intervention: DOACs; (4) comparison: VKAs; (5) outcomes: one of the followings: thrombus resolution, stroke or systemic embolism (SSE), stroke clinically relevant bleeding or all bleeding events. Moreover, abstracts that were not published as a full paper were also being considered for inclusion if they meet the selection criteria.

### 2.3 Data Extraction and Quality Assessment

Two researchers independently reviewed the eligibility of identified articles. The information which we extracted from each study included: first author’ name, year of publication, sample size, location, patients’ characteristics, and outcome data.

The quality of non-randomized studies was evaluated by modified Newcastle-Ottawa (NOS) scale [15]. The maximum of nine points were awarded to each study. And if a study was scored more than 5 points, it was considered as high quality.

### 2.4 Statistical Analysis 

The risk ratio (RR) and 95% confidence intervention (95% CIs) was used to calculate the dichotomous variables. Heterogeneity among the included studies was assessed using Cochrane Q chi-square and *$I^{2}$* statistic [16]. A *p *value < 0.1 or *$I^{2}$* >50% indicated the evidence of heterogeneity [16]. Summarized effect sizes were computed by a fixed-effects model [17] when no significant heterogeneity was identified; otherwise, a randomized-effects model [18] was applied. Considering the difference in study quality among the included studies, a sensitive analysis to account for potential bias will be performed. We also carried out subgroup analysis according to the use of antiplatelet and treatment switching to explore whether these variables have an impact on the overall estimate. Begg’s [19] and Egger’s test [20] were used to assess the publication bias. All statistical analyses were performed by STATA version 12.0 (Stata Corporation, College Station, TX, USA).

### 2.5 Meta-Regression Analyses

We hypothesized that various clinical variables might have affected the results of included studies; these included study design: prospective or retrospective cohort, duration of follow-up: <1 year or ≥1years, sample size: <100 or ≥100, concomitant antiplatelet medication, primary cause of LVT: MI or mixed reasons, and types of DOACs: apixaban <50% or apixaban ≥50%. In order to identify whether the different results were influenced by the variables, we performed meta-regression analyses. In this model, outcomes were regarded as a dependent variable (y) and the covariates described above as independent variables (χ).

## 3. Result

### 3.1 Study Selection

**Fig. 1.**
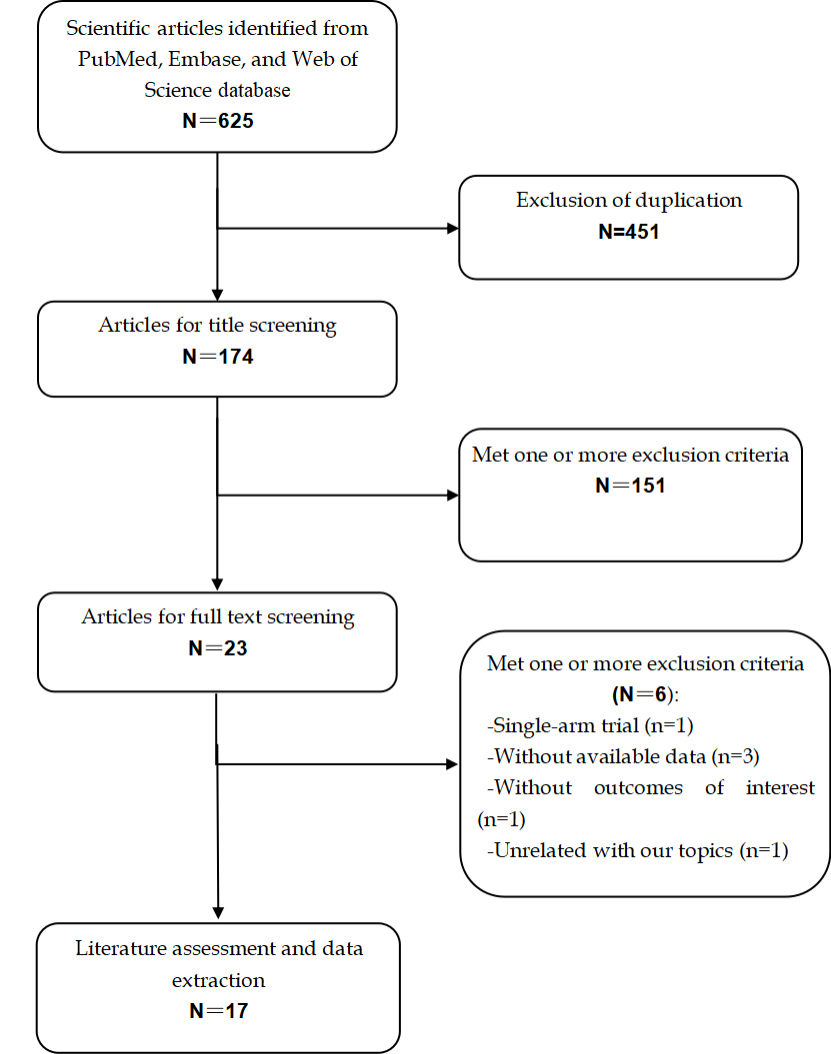
**Study selection process**.

The search strategy identified 625 articles, and 451 duplicates were excluded. Then 151 publications were excluded by screening the abstract/title. The remaining 23 studies were retrieved for full-text review, and 6 studies were removed due to the following reasons: 3 did not provide available data, 1 was a single-arm study, 1 provided outcomes out of our interest, and 1 was unrelated with our topics. Finally, seventeen studies [8, 9, 21, 22, 23, 24, 25, 26, 27, 28, 29, 30, 31, 32, 33, 34, 35] with 2683 patients were included for qualitative synthesis (Fig. 1).

### 3.2 Characteristics of the Included Studies

The characteristics of included studies is presented in Table 1 (Ref. [8, 9, 21, 22, 23, 24, 25, 26, 27, 28, 29, 30, 31, 32, 33, 34, 35]). The samples varied in size from 28 and 1129. Seven of the 15 studies were conducted in USA, four in UK, one in Switzerland, one in France, one in Malaysia, one in Egypt, one in Israel and one in Portugal. All the studies were retrospective cohort studies [8, 9, 21, 22, 23, 24, 25, 26, 27, 29, 30, 31, 32, 33] except for two RCTs [34, 35] and one prospective cohort study [28]. The patients’ mean age in each study ranged from 52.3 years to 69.7 years, and 75.82% of the enrolled patients were male patients. Six hundred and seventy-seven patients received the treatment of DOACs and 1955 patients received VKAs. Hypertension was presented in 610 (22.74%) patients, and diabetes in 431 (16.06%) patients. Seven studies with 289 patients (10.77%) reported the treatment switching of DOACs between the two groups. The most common cause of treatment switching was convenience or cost. Among the DOAC groups, apixaban (51.32%) was the most frequently prescribed, followed by rivaroxaban (39.85%), dabigatran (8.57%), and edoxaban (0.26%); whereas, warfarin (97.63%) was the predominantly prescribed in the VKAs group. A concomitant antiplatelet medication was prescribed in over half of patients, although dual antiplatelet therapy was less frequently used. The antiplatelet therapy among these studies included aspirin, clopidogrel and P2Y12 inhibitors (ticagrelor or prasugrel).

**Table 1.** **Demographic and clinical characteristics of the patients at baseline**.

Study	Treatment regimen	Age (mean ± SD, y)	Female (n, %)	HTN (n, %)	DM (n, %)	AF (n, %)	LVEF, % (mean ± SD)	Ischemic CM (n, %)	AAS (n, %)	NOS score
Daher J [8]	DOAC (n = 17)	57 ± 14	3 (18)	10 (59)	2 (12)	NR	41 ± 8	15 (88)	10 (59)	4
	VKA (n = 42)	61 ± 13	7 (17)	17 (41)	9 (21)	NR	36 ± 12	36 (74)	28 (66)	
Jones DA [9]	DOAC (n = 41)	58 ± 14	8 (20)	23 (61)	7 (18)	NR	34 ± 10	NR	NR	5
	VKA (n = 60)	60 ± 14	9 (15)	22 (36)	10 (17)	NR	35 ± 9	NR	NR	
Robinson AA [21]	DOAC (n = 121)	58 ± 15	27 (22)	86 (71)	36 (30)	30 (25)	28 ± 14	66 (55)	56 (46)	6
	VKA (n = 236)	58 ± 15	66 (28)	177 (75)	92 (39)	45 (19)	28 ± 12	148 (63)	109 (46)	
Bass M [22]	DOAC (n = 180)	66	55 (31)	NR	NR	NR	NR	77 (43)	111 (62)	6
	VKA (n = 769)	63	224 (29)	NR	NR	NR	NR	443 (58)	352 (46)	
Jaidka A [23]	DOAC (n = 12)	57 ± 9	3 (25)	2 (12)	1 (8)	NR	37 ± 10	0 (0)	9 (75)	5
	VKA (n = 37)	61 ± 12	9 (24)	18 (49)	7 (19)	NR	20 ± 21	3 (8)	33 (89)	
Gama F [24]	DOAC (n = 13)	69 ± 12	NR	NR	NR	NR	NR	NR	NR	4
	VKA (n = 53)	69 ± 12	NR	NR	NR	NR	NR	NR	NR	
Cochran JM [25]	DOAC (n = 14)	52	3 (21)	NR	7 (50)	NR	NR	7 (50)	NR	5
	VKA (n = 59)	62	14 (24)	NR	23 (39)	NR	NR	36 (61)	NR	
Iqbal H [26]	DOAC (n = 22)	62 ± 13	2 (9)	9 (41)	19 (86)	NR	31 ± 13	18 (82)	9 (41)	5
	VKA (n = 62)	62 ± 14	7 (11)	18 (29)	19 (31)	NR	35 ± 13	55 (89)	39 (65)	
Guddeti RR [27]	DOAC (n = 19)	61 ± 13	4 (21)	15 (79)	3 (16)	4 (21)	25 (20–40)	10 (53)	11 (58)	5
	VKA (n = 80)	61 ± 12	25 (21)	61 (76)	34 (43)	18 (23)	25 (20–35)	48 (60)	54 (68)	
Alizadeh M [28]	DOAC (n = 38)	NR	NR	NR	NR	NR	NR	NR	NR	4
	VKA (n = 60)	NR	NR	NR	NR	NR	NR	NR	NR	
Lim CW [29]	DOAC (n = 5)	55 ± 9.6	2 (40)	3/2	3/2	NR	30 ± 10	NR	NR	5
	VKA (n = 18)	55 ± 9.6	5 (26)	10/8	9/9	NR	30 ± 10	NR	NR	
Yunis A [30]	DOAC (n = 64)	NR	NR	NR	NR	NR	NR	NR	NR	6
	VKA (n = 200)	NR	NR	NR	NR	NR	NR	NR	NR	
Willeford A [31]	DOAC (n = 22)	54	5 (22)	8 (36)	4 (18)	3 (14)	NR	5 (23)	NR	4
	VKA (n = 129)	56	25 (19)	54 (42)	37 (29)	24 (19)	NR	34 (26)	NR	
Ali Z [32]	DOAC (n = 32)	59 ± 12	6 (19)	NR	12 (38)	9 (28)	23 ± 9	NR	NR	5
	VKA (n = 60)	58 ± 16	11 (18)	NR	18 (30)	18 (30)	23 ± 11	NR	NR	
Durrer‑Ariyakuddy K [33]	DOAC (n = 20)	63	5 (25)	NR	NR	NR	32 ± 12	NR	NR	4
	VKA (n = 33)	63	9 (27)	NR	NR	NR	32 ± 12	NR	NR	
Abdelnabi M [34]	DOAC (n = 39)	49.6 ± 12.5	NR	21 (53)	21 (53)	NR	NR	NR	NR	NA
	VKA (n = 79)	49.6 ± 12.5	NR	42 (54)	42 (54)	NR	NR	NR	NR	
Alcalai R [35]	DOAC (n = 18)	55.5 ± 12.9	5 (28)	7 (39)	7 (39)	NR	NR	NR	NR	NA
	VKA (n = 17)	58.8 ± 10.2	2 (12)	7 (41)	9 (53)	NR	NR	NR	NR	

**Abbreviation**: SD, standard deviation; HTN, hypertension; DM, diabetes mellitus; AF, atrial fibrillation; LVEF, left ventricular ejection fraction; CM, cardiomyopathy; AAS, acetylsalicylic acid; DOAC, direct oral anticoagulants; VKA, vitamin-K antagonists; NR, not reported; NA, not available.

Quality assessment of cohort studies showed that these studies had a NOS score between 4 and 6, indicating a low or high quality. The reason for five studies with low quality was that several important factors were not well-balanced between the DOAC and VKA groups, or the follow-up time was not long enough to assess the outcomes.

### 3.3 Stroke or Systemic Embolism 

**Fig. 2.**
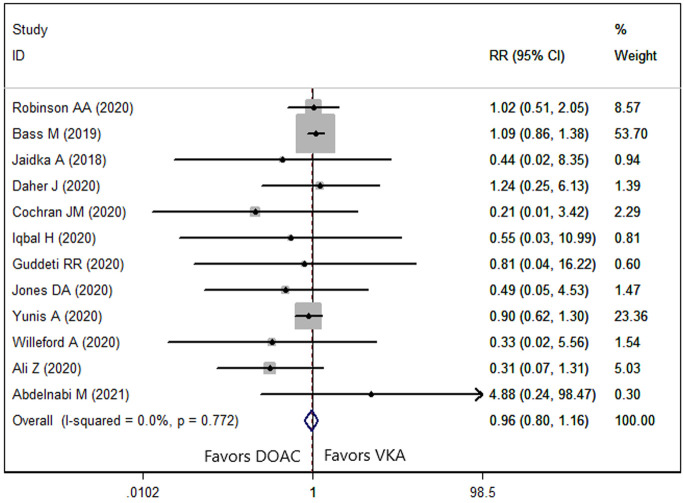
**Forest plot showing the comparison between DOACs and VKAs in the risk of SSE**.

Twelve studies [8, 9, 21, 22, 23, 25, 26, 27, 30, 31, 32, 34] reported the data of SSE. The prevalence of SSE in patients treated with DOAC and VKA was 17.07% and 21.62%, respectively. Pooled estimate using a fixed-effects model (*p* = 0.772, *$I^{2}$* = 0.0%) suggested that DOACs had a comparable SSE rate with VKAs (RR = 0.96, 95% CI: 0.80, 1.16; *p* = 0.677) (Fig. 2). We conducted a meta-regression analysis for clinical variables (study design, sample size, duration of follow-up, concomitant antiplatelet medication, primary cause of LVT, types of DOACs). Results indicated that all these variables had no impact on the SSE risk (Table 2).

**Table 2.** **Results of meta-regression analysis for the impact of clinical and demographic data on the outcomes**.

	SSE			Thrombus resolution			Bleeding events		
	Coefficient	95% CI	*p*	Coefficient	95% CI	*p*	Coefficient	95% CI	*p*
Study design	4.47	–0.05,8.98	0.051	0.56	–0.24, 1.35	0.142	2.32	0.23, 4.42	0.037
Sample size	0.51	–1.03, 2.05	0.409	0.28	–0.36, 0.93	0.334	0.31	–1.65, 2.28	0.678
Duration of follow-up	–0.18	–0.73, 0.38	0.431	0.01	–0.62, 0.62	0.998	–0.47	–2.78, 1.83	0.596
Concomitant antiplatelet medication	–1.43	–7.97, 5.09	0.575	–0.45	–1.35, 0.44	0.270	0.08	–2.51, 2.66	0.939
Primary cause of LVT	0.29	–2.42, 3.00	0.781	–0.03	–0.66, 0.61	0.920	1.11	–1.34, 3.55	0.278
Types of DOACs	–0.09	–2.67, 2.47	0.924	0.14	–0.21, 0.48	0.380	0.44	–1.35, 2.23	0.532

**Abbreviation**: SSE, stroke or systemic embolism; LVT, Left ventricular; DOACs, direct oral anticoagulants; 95% CI, 95% confidence interval.

**Fig. 3.**
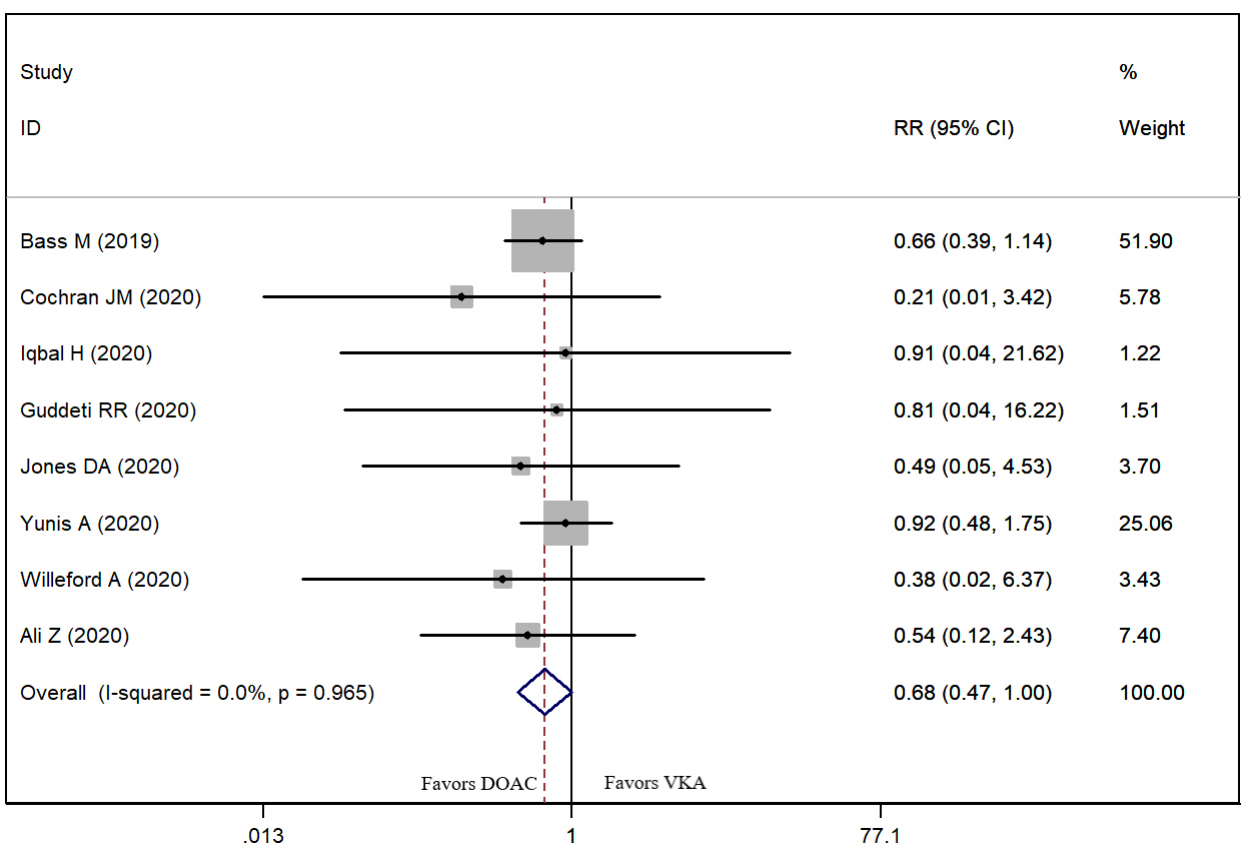
**Forest plot showing the comparison between DOACs and VKAs in the risk of stroke**.

Eight studies presented the data on stroke [9, 22, 25, 26, 27, 30, 31, 32]. The prevalence of stroke in patients treated with DOAC and VKA was 6.85% and 10.78%, respectively. The pooled data showed that, DOACs significantly reduced the stroke risk as compared to VKAs (RR = 0.68, 95% CI: 0.47, 1.00; *p* = 0.048) (Fig. 3).

### 3.4 Thrombus Resolution

**Fig. 4.**
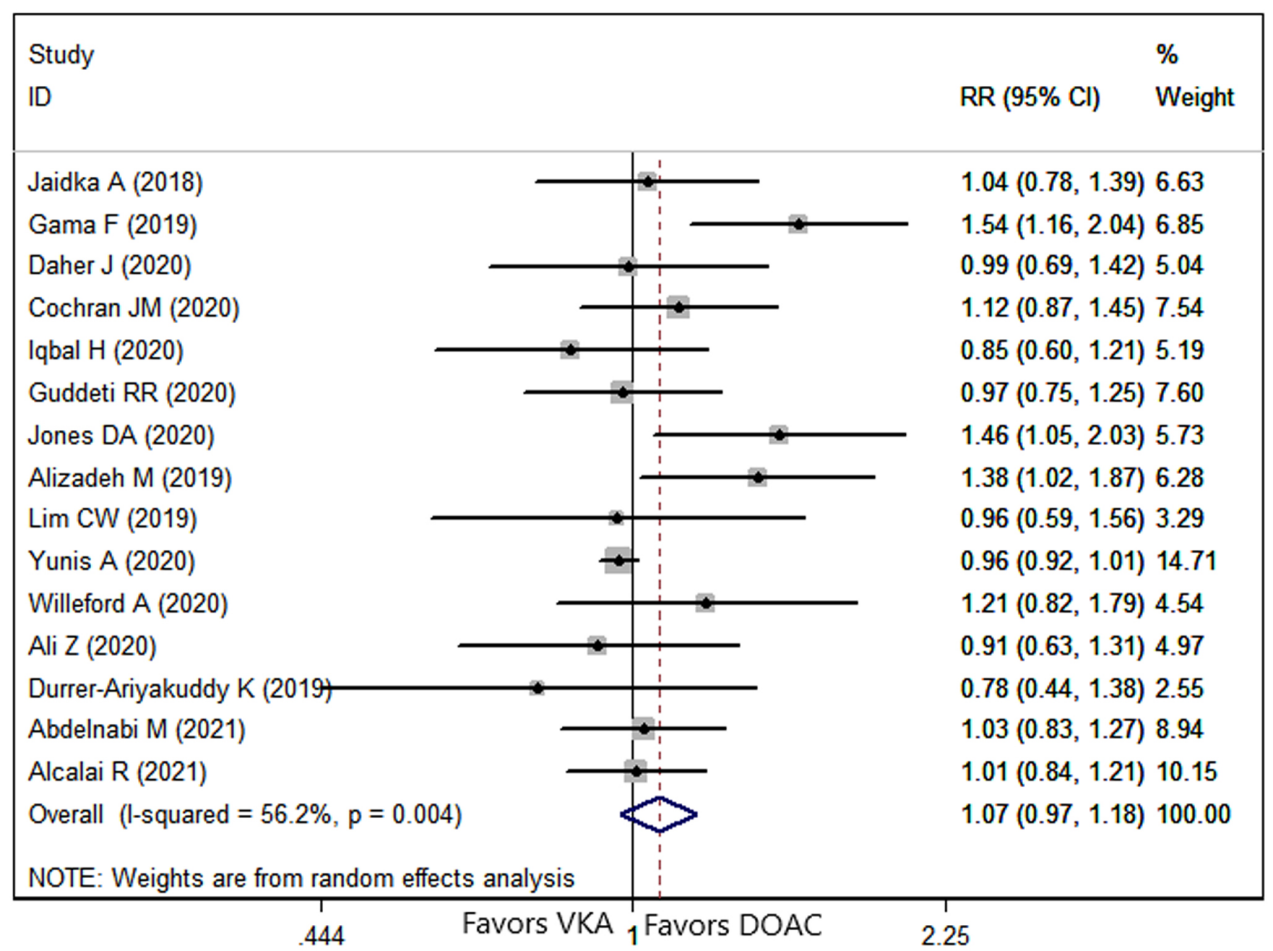
**Forest plot showing the comparison between DOACs and VKAs in the thrombus resolution**.

Fifteen studies [8, 9, 23, 24, 25, 26, 27, 28, 29, 30, 31, 32, 33, 34, 35] reported the data of thrombus resolution. The prevalence of thrombus resolution for patients in DOAC group was 76.42% compared with 72.72% for patients in VKA group. Pooled data demonstrated that, the resolution rate was similar between the two groups (RR = 1.07, 95% CI: 0.97, 1.18; *p* = 0.193) (Fig. 4).

The meta-regression analysis of thrombus resolution demonstrated that none of these variables tested were significantly associated with the outcome of thrombus resolution (Table 2).

### 3.5 Bleeding Events

**Fig. 5.**
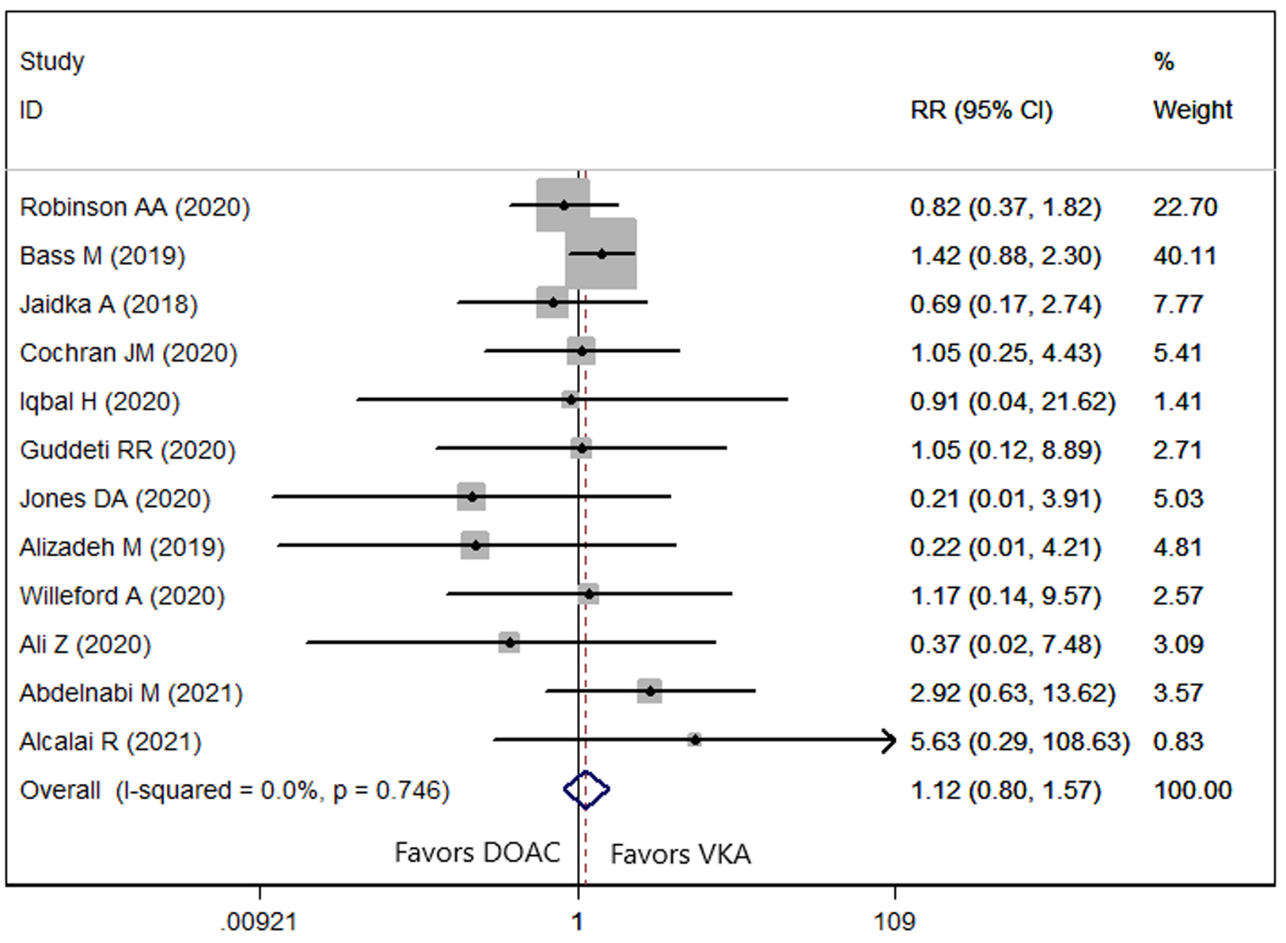
**Forest plot showing the comparison between DOACs and VKAs in the bleeding events**.

Twelve studies [9, 21, 22, 23, 25, 26, 27, 28, 31, 32, 34, 35] reported the data of bleeding events. The prevalence of bleeding events in patients treated with DOAC and VKA was 6.81% and 7.46%, respectively. No significant difference in identified among patients in the two groups (RR = 1.12, 95% CI: 0.80, 1.57; *p* = 0.386) (Fig. 5). There was no evidence of heterogeneity across included studies (*p* = 0.746, *$I^{2}$* = 0.0%). The meta-regression analysis was presented in Table 2, showing that none of these variables had any impact on the bleeding events.

Seven studies reported the data of clinically relevant bleeding events [9, 23, 26, 27, 28, 31, 32]. The rate of clinically relevant bleeding events in DOAC and VKA groups was 2.73% and 6.99%, respectively. Pooled estimate showed a lower rate of clinically relevant bleeding events in the DOAC group (RR = 0.49, 95% CI: 0.23, 1.03; *p* = 0.060); however, this difference did not reach the statistically significant.

### 3.6 Subgroup Analysis Based on the Use of Antiplatelet and Treatment Switching

The results of subgroup analysis are presented in Table 3. It showed that patients with different treatment regimens had comparable outcomes (SSE, thrombus resolution, bleeding events and clinically relevant bleeding events).

**Table 3.** **Subgroup analysis based on the use of antiplatelet and treatment switching**.

		SSE	Thrombus resolution	Bleeding events	CRBE
Use of antiplatelet					
	Yes	0.72 (0.44, 1.19); *p* = 0.198	1.04 (0.88, 1.23); *p* = 0.625	0.98 (0.60, 1.61); *p* = 0.932	0.48 (0.21, 1.11); *p* = 0.086
	No	1.03 (0.85, 1.25); *p* = 0.762	1.06 (0.98, 1.14); *p* = 0.125	1.28 (0.81, 2.02); *p* = 0.287	0.52 (0.10, 2.70); *p* = 0.438
Treatment switching					
	Yes	0.89 (0.48, 1.66); *p* = 0.723	1.07 (0.97, 1.19); *p* = 0.162	1.00 (0.52, 1.94); *p* = 0.994	-
	No	0.97 (0.80, 1.18); *p* = 0.764	1.03 (0.95, 1.12); *p* = 0.426	1.17 (0.79, 1.73); *p* = 0.426	-

**Abbreviation**: SSE, stroke or systemic embolism; CRBE, clinically relevant bleeding events.

### 3.7 Sensitive Analysis

In order to detect potential bias introduced by the study quality, RRs were calculated by studies with high quality and were compared to the results obtained from all studies (Table 4). Interesting, a remarkable change was observed with the stroke, which showed a similar risk of stroke between the two groups (RR = 0.69, 95% CI: 0.47, 1.02; *p* = 0.06).

**Table 4.** **Sensitive analysis of the outcome by applying different study quality**.

		RR	95% CI	*p* value
SSE				
	All studies	0.98	0.80, 1.16	0.677
	High-quality studies	0.97	0.80, 1.17	0.728
Stroke				
	All studies	0.68	0.47, 1.00	0.048
	High-quality studies	0.69	0.47, 1.02	0.06
Thrombus resolution				
	All studies	1.07	0.97, 1.18	0.193
	High-quality studies	1.00	0.93, 1.08	0.94
Bleeding events				
	All studies	1.17	0.82, 1.65	0.386
	High-quality studies	1.19	0.84, 1.70	0.327
Clinically relevant bleeding events				
	All studies	0.49	0.23, 1.03	0.060
	High-quality studies	0.47	0.20, 1.10	0.081

**Abbreviation**: SSE, stroke or systemic embolism; 95% CI, 95% confidence interval.

### 3.8 Publication Bias

The test for publication bias revealed no evidence of publication bias across the studies (Egger’s test: *p* = 0.127; Begg’s test: *p* = 0.839).

## 4. Discussion

The purpose of this meta-analysis was to compare the efficacy and safety of DOACs and VKAs for patients who were diagnosed with LVT. Our findings suggested that DOACs showed comparable effect in prevention of SSE and LVT resolution as compared to VKAs. The prevalence of stroke was significantly lower in DOAC users than that in VKA users; however, this effect was not observed among studies with high-quality. Moreover, DOACs showed comparable risk of bleeding events or clinically relevant bleeding events in the treatment of LVT as compared with VKAs.

There have been several meta-analyses that compared the effects of DOACs with VKAs for LVT [36, 37, 38, 39, 40]. Our study expends on the prior studies in providing more significant evidence for the efficacy and safety assessment of the two treatment regimens in LVT. First, this study had enlarged sample size than the prior reviews, which improved the statistical power to assess treatment effects. In the present study, we included 17 studies with 2683 patients, which were prospective/retrospective cohort studies or RCTs. Whereas, in the previous reviews [36, 39], they only included 5 or 6 studies, and the sample size in their studies ranged from 700 to 1104. Second, meta-regression analysis was carried out to evaluate whether several variables had impact on the outcomes. This was not done in the previous meta-analysis. Third, we conducted sensitivity analysis to explore whether the overall estimate would be biased by the study quality. Fortunately, no notable difference in these outcomes was identified in the analysis, which confirmed the reliability of our findings. Fourth, subgroup analysis was conducted according to the use of antiplatelet and treatment switching, which found no significant difference in the subgroup analysis. Overall, the enhanced sample size, meta-regression analysis and sensitivity analysis ensure the credible and robust of our findings.

In the present study, the pooled results showed a similar SSE risk of DOACs with VKAs in patients with LVT. This was in line with the findings of prior studies [8, 25, 26, 30, 37, 38]. Michael F, *et al*. [37] performed a meta-analysis to compare the safety and efficacy of DOACs versus VKAs for LVT and found a comparable effect in SSE between the two treatments (odds ratio (OR) = 0.83, 95% CI: 0.53, 1.33; *p* = 0.45). Similarly, in another meta-analysis of Saleh Y, *et al*. [38], the authors observed no significant difference between rivaroxaban and VKAs in terms of SSE (OR = 0.73, 95% CI: 0.24, 2.22; *p* = 0.58). Overall, our findings were in agreement with that of the prior meta-analysis, which demonstrated a similar risk of SSE between the two treatments.

In contrast, one of the largest cohort studies by Robinson AA, *et al*. [21] showed contrast results of the SSE risk between the two treatments. In that study, 514 patients with LVT were recruited from 3 tertiary care academic medical centers. Of them, 300 were assigned to warfarin group and 185 were to DOAC group [21]. In the unadjusted analysis, patients in DOAC group experienced a significantly higher prevalence of SSE than those in warfarin group (hazard ratio (HR) = 2.13, 95% CI: 1.31, 5.57; *p* = 0.01) [21]. In the multivariable analysis, the DOAC still showed increased risk of SSE than warfarin (HR = 2.64, 95% CI: 1.28, 5.43; *p* = 0.01), which suggested that DOAC had weaker effect than warfarin in decreasing the risk of SSE. However, in that study, more than 15% of patients included in their analysis had switch therapy, which made it difficult to assess the true risk difference across the two treatment regimens.

In this study, the prevalence of thrombus resolution was comparable between the two treatments. This was in accordance with the findings of previous studies [23, 25, 26, 31, 34, 35]. Abdelnabi M, *et al*. [34] performed a prospective, multicenter, randomized trial in 79 patients in Egypt and Bulgaria. In that study, 39 patients were randomly assigned into rivaroxaban group and 40 patients into warfarin group. At the end of 1, 3, and 6 months, 28 (71.79%), 30 (76.92%), and 34 (87.17%) patients in the rivaroxaban group occurred complete LVT resolution, as compared to 19 (47.5%), 27 (67.5%), and 32 (80%) patients in the warfarin group [34]. This did not differ significantly between the two groups (adjusted *p* values after Bonferroni correction = 0.084, 0.700, and 0.700, respectively). This similar effect of thrombus resolution was also reported by another prospective, randomized, multicentre open-label trial [35]. In that study, the authors identified a higher prevalence of complete resolution of thrombus with apixaban (94.1%, 16/17) over warfarin (93.3%, 14/15) at 3-month follow-up. However, the difference did not reach statistical significance (*p* = 1, superiority). Although a high rate of complete resolution of visible LVT was found in that study, the authors pointed out that their results was still limited by the relatively small sample size, use of large margin for non-inferiority, and lack of sufficient statistical power [35]. On the other hand, some other studies found a higher prevalence of LVT resolution with DOACs when compared to VKAs [9, 40]. Jones DA, *et al*. [9] reported a significantly higher rate of thrombus resolution in DOAC (82%) group than that in warfarin (64.4%) group at 1 year. And this result persisted even after the adjustment of baseline variables (OR = 1.8, 95% CI: 1.2, 2.9) [9]. The authors did not give any explanations for the contrast results. However, their study was designed with convenience sampling, which might lead to selection bias. Chen R, *et al*. [40] found that DOACs had a significantly higher prevalence of thrombus resolution than VKAs in patients with MI (RR = 0.57, 95% CI: 0.38, 0.84; *p* = 0.005). They explained that the discrepancy result might be caused by the increased thrombotic burden after MI [40].

In terms of the bleeding events, DOACs had similar risk of bleeding events or clinically relevant bleeding events in patients with LVT, as compared to VAKs. This was accordance with the previous studies [21, 22, 23, 25]. Cochran JM, *et al*. [25] reported a similar rate of bleeding events with DOACs (14%) compared with VKAs (14%). Similarly, Jaidka A, *et al*. [23] reported a comparable rate of major bleeding (0% vs 8.3%, *p* = 0.549) and minor bleeding (16.2% vs 16.7%, *p* = 0.971) events with VKAs in a study of 64 LVT patients. Our results regarding the risk of bleeding events or clinically relevant bleeding events were consistent even after performing the sensitive analysis. However, in another meta-analysis of 5 studies, the authors reported a reduced prevalence of bleeding events in DOAC group than the VKA group (OR = 0.49, 95% CI: 0.26, 0.90; *p* = 0.02) [36]. The discrepancy result might be caused by the small sample size for data analysis and the varied criteria for reporting bleeding events included in that review.

This study has several potential limitations. First, the sample size in some included studies was relatively small, which might result in certain selection bias. Second, most of the studies were retrospective cohort studies. Despite observational studies provide less robust level of evidence as RCTs since they are more likely to selection bias, the high-quality observational studies can importantly contribute to the totality of evidence for the benefits and risks of an intervention because they often have less restrictive inclusion criteria and treatment. Moreover, they can reflect the real-world. Lastly, there were differences in regimen and dosage of DOACs among the included studies, which might undermine its comparability with VKAs in the data analysis.

## 5. Conclusions

In conclusion, our results suggested that DOACs had similar effect with VKAs in the prevention of LVT in terms of SSE risk and thrombus resolution. Moreover, the incidences of bleeding events or clinically relevant bleeding events were also comparable between the two treatment regimens.
